# Combining international survey datasets to identify indicators of stress during the COVID-19 pandemic: A machine learning approach to improve generalization

**DOI:** 10.1192/j.eurpsy.2022.951

**Published:** 2022-09-01

**Authors:** M. Adamson, E. Zhao, D. Xia, E. Colicino, M. Monaro, R. Hitching, O. Harris, M. Greenhalgh

**Affiliations:** 1 Stanford University School of Medicine, Department Of Neurosurgery, Stanford, United States of America; 2 Veterans Affairs Palo Alto Health Care System, Rehabilitation Service, Palo Alto, United States of America; 3 stanford University, Department Ofcomputer Science And Engineering, Stanford, United States of America; 4 Icahn School of medicine at Mount Sinai, Environmental Medicine And Public Health, New york, United States of America; 5 university of Padua, General Psychology, Padua, Italy

**Keywords:** machine learning, Stress, Covid-19, international

## Abstract

**Introduction:**

The magnitude and exceptional opportunity to research the psychological distress of shelter in place resulted in a publication frenzy on a smorgasbord of research studies of variable scientific robustness. Confinement, fear of contagion, social isolation, financial hardship, etc. equated to stratospheric stress levels. The decline in protective factors as a function of quarantine anecdotally reflected historic rates of anxiety and depression.

**Objectives:**

In this study, we combined 12 variegate datasets and developed an algorithm to build a model to identify key predictors of pandemic-related stress with high accuracy and generalizability.

**Methods:**

This study reports on existing published data. We first describe the International (Adamson et al., 2020) and then the Italian dataset (Flesia et al., 2020). The time-frame (first wave of lockdown), method (survey), measurement tool (Perceived Stress Scale), and outcome measures were extremely similar to enable consolidation of datasets (see Figure1). The Flesia et al., (2020) data set was integrated into the Adamson et al., (2020) dataset as the first step towards data validation construction of the ML predictive model.

**Results:**

We aim to demonstrate the strength of combining cross-cultural datasets, and the applicability of ML algorithms to facilitate the process and generate a predictive model that identifies and validates key predictors of pandemic-related stress and accommodates for interaction with demographic, cultural, and other mitigating factors while concurrently having high generalizability.

**Conclusions:**

We believe our model provides clinicians, researchers, and decision-makers with evidence to investigate the moderators and mediators of stress, and introduce novel interventions to mitigate the long-term effects of the COVID-19 pandemic.

**Disclosure:**

No significant relationships.

